# Vanderbilt University, Department of Dentistry

**Published:** 1882-03

**Authors:** W. H. Morgan

**Affiliations:** Dean.


*Vanderbilt University, Department of Dentistry.*—The Chapel of Vanderbilt University was filled on the night of February 24th, to witness the commencement exercises of the Department of Dentistry. The address on the part of the class was delivered by M. J. Lunquest, of Alabama, and the address on the part of the Faculty by Prof. R. R. Foreman. The degree of D. D. S. was conferred by the Chancellor on the following gentlemen :

Thomas H. Lipscomb, of Tenn.; Wm. H. Kemper, M. D., Mo.; Stephen R. Jordan, Ga.; James W. Hambright, Ga.; John M. Powell, Ark.; Edward W. Tillett, Tenn.; William J. Flanders, Ga.; William A. Flemister, Ga.; John H. Magruder, Miss.; Francis H. McAnnally, Ala.; Marques J. Lunquest, Ala.; James Allen Barr, Ky.; Thomas M. Allen, Ala.; David R. Stubblefield, Tenn.

Number Matriculating, 33. One lady among them.

W. H. Morgan, *Dean.*

